# Multi‐Axis Fatigue Experimentation System of Intramedullary Implants for Femur and Tibia

**DOI:** 10.1002/jor.24545

**Published:** 2019-12-10

**Authors:** Mikko Kanerva, Tuomas Pärnänen, Jarno Jokinen, Juha Haaja, Antti Ritvanen, Dietrich Schlenzka

**Affiliations:** ^1^ Faculty of Engineering and Natural Sciences Tampere University P.O.B 589 FI‐33014 Tampere Finland; ^2^ Orton Orthopaedic Hospital and Research Institute Orton FI‐00280 Helsinki Finland; ^3^ Synoste Oy, Metsänneidonkuja 6 FI‐02130 Espoo Finland

**Keywords:** implant, fatigue, multi‐axis loading, consolidation phase, finite element analysis

## Abstract

Current designs of leg‐lengthening implants have faced serious failures due to inadequacies in the mechanical design. The failure typically is the result of fatigue induced by a combined loading condition with axial and shear components acting in the tubular body of the implant. One of the reasons leading to the failure is improper verification testing for the design of the fatigue limit. The current test standards for pre‐clinical design phases of nail implants are relatively straightforward and widely accepted yet cannot produce the three‐dimensional stress state representative of the anticipated operation in a patient during the consolidation phase. This work introduces a major improvement toward a method for verifying fatigue life of tubular as well as solid implants under combined torque, axial load, and bending. The report describes a new loading fixture, a calibration method, and compares the qualification results of finite element simulation analyses and experimental measurements during cyclic loading tests. The findings state that the fixture produces controlled multi‐axial loadings to study varied osteotomy locations, quasi‐static strength and fatigue of intramedullary implants at an intermediate, 2 Hz, cycle rate. © 2019 The Authors. *Journal of Orthopaedic Research*® published by Wiley Periodicals, Inc. on behalf of Orthopaedic Research Society. J Orthop Res 38:984‐995, 2020

## Graphical abstract

Fatigue in intramedullary implants causes severe complications due to the failure of implants and related operation during the consolidation phase. This work succeeds in developing a test fixture and procedure for inducing three‐dimensional loading in order to define the fatigue life of an implant. The fatigue can be analyzed for various osteotomy locations and for various load spectra based on the estimates made for a patient or patient group. The fixture induces reliable loadings at cycle rates up to 2 Hz.

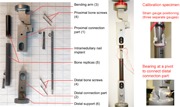

The recent development of metal alloys, the understanding of stress tri‐axiality and the improvements in designing surgeries individually for patients have led to new designs of implants. These implants are used for trauma patients as well as re‐building of skeletal anomalies, such as an inherent imbalance in limb lengths. The leg‐lengthening implants represent a specific type of implant capable of elongating in the implant's long‐axis direction. The first systems for femoral leg‐lengthening relied on external actuation. Various concepts have been developed for telescopic nails, which are intramedullary implantable and actuated by either manual ratcheting,^1^ electro‐motorized power unit,^2^ kinetic distractors,^3^ magnet‐gearbox unit,^4^ or even hydraulic power source.^5^ Due to the sheer size of the human femur and its internal microstructure, the size, namely diameter, of the implant is strictly limited. In order to fit required actuation mechanisms inside the implant body, the load‐carrying materials are at their limits in terms of the fatigue limit and fracture toughness.^6^, ^7^, ^8^

A nail implant, either solid or telescopic, experiences mechanical loading during the operational phases, simply the lengthening and consolidation phases. The different sources of mechanical loads are basically well‐known:

- Bodyweight of patient;
- Muscle contraction‐induced loading;
- Transient loads during dynamic loading, for example, when the patient is walking;
- Lengthening loads due to the soft tissue tension activated by implant work.

The most demanding load types are the muscle contraction‐induced loads and transient loads when walking. The patient's body weight is subjected to the nail structure through the bone (femur) and the fixing point. The bone, when intact, itself experiences the body weight acting between its proximal and distal end. Due to the typical shape of the proximal end at antercurvatum, the force vector of body weight is offset from the intended plane of nail implantation. The offset induces a bending moment along the long axis (i.e., anatomic axis) of the bone and a nail. Additionally, due to the combination of the offset and angular alignment of the force vector with respect to the long axis of the bone, torque is induced over the long axis. Of these three load components, meaning the axial force, bending moment and torque, the axial force component is primarily carried by the nail after osteotomy. Therefore, the axial component is of main importance during mechanical testing.

Currently, the ASTM F1264 standard provides the test methods for studying the mechanical performance of intramedullary fixation devices and defines the test procedure for four‐point bending tests.^9^ The existing test methods are suited for studying torsional and relative bending performance separately in a single load mode at a time. The four‐point bending test fixture induces localized surface loads and results in an unrepresentative loading‐deformation especially in an implant that is hollow. Additionally, the four‐point bending test does not induce any axial loading—this load component is important, for example, for limb lengtheners. Clearly, the full three‐dimensional (3D) loading during testing introduces a more realistic load scheme for the fixation devices. In this study, we pursue to improve the testing of femur and tibia mounted implants so that the multi‐axis load conditions can be accounted for. The challenges are the reproduction of a 3D load‐combination into an implant connected to the test fixture. Moreover, a controlled load sequence must be produced at different load rates to practically produce the entire fatigue load spectrum existing due to the patient walking in real‐life events. The principles of load components acting on the femur during the patient's movement and the fundamental difference between the loads of standard four‐point bending and those of a multi‐axis fixture are illustrated in Figure 1.

**Figure 1:**
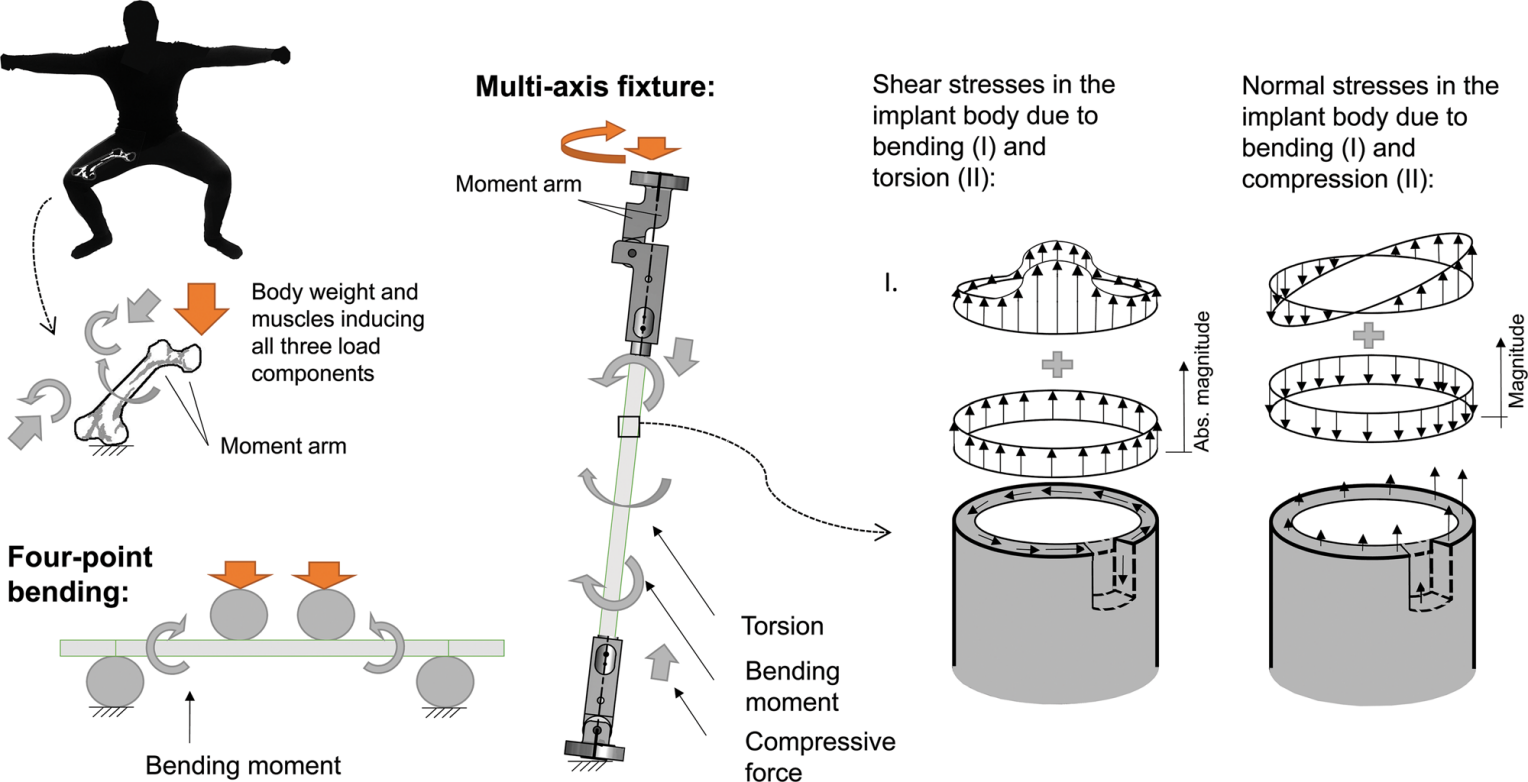
Schematic illustration of the load components acting on the femur during the patient's movement. The load introduction by the standard four‐point bending (in the lower‐left corner) and a multi‐axis fixture (in the middle). Typical distributions of shear and normal stresses inside a tubular implant body due to complete loading is presented on the right side. [Color figure can be viewed at wileyonlinelibrary.com].

## MATERIALS AND METHODS

### Multi‐Axis Loading Fixture

**Figure 2:**
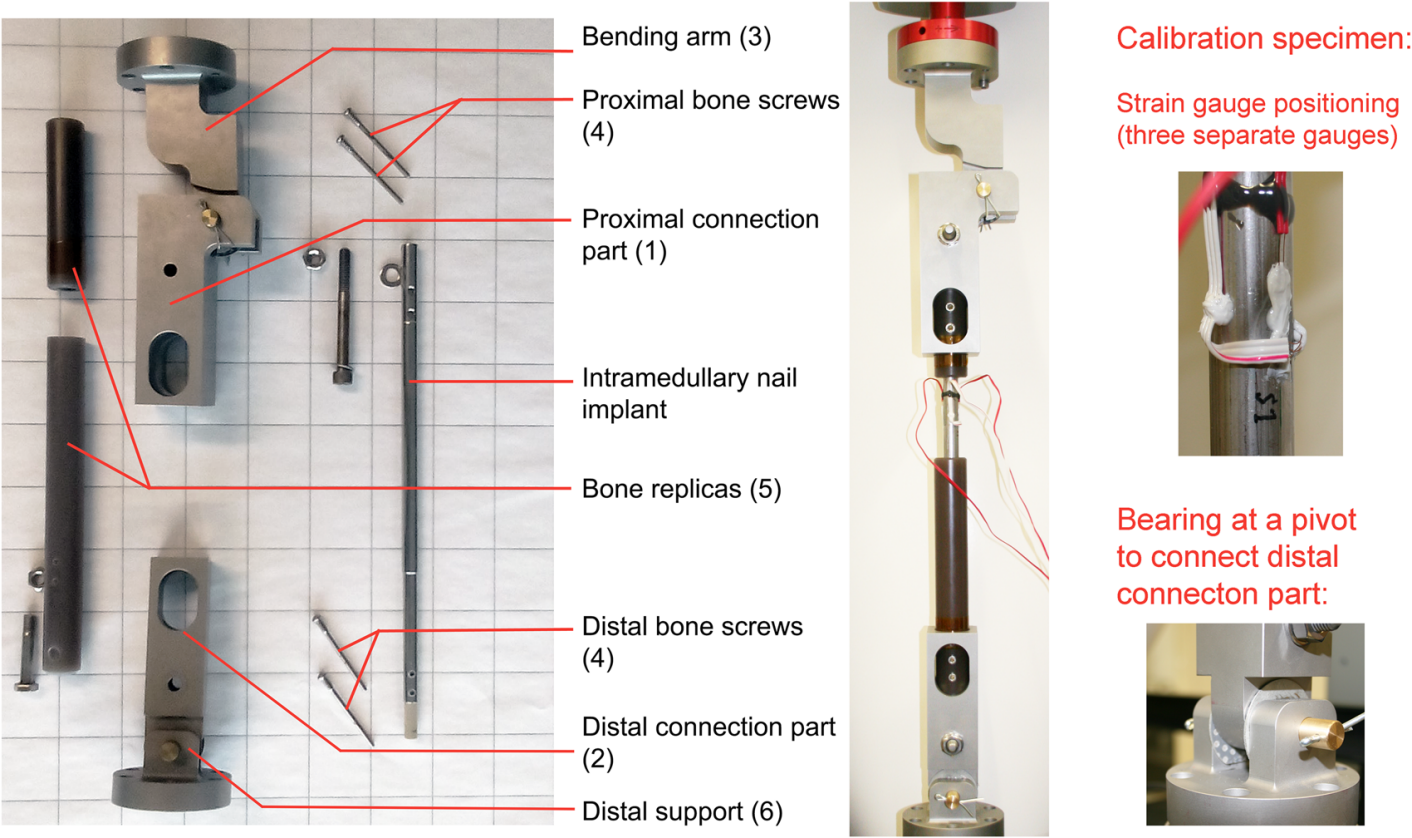
Multi‐axis loading fixture: part list and the description of a calibration specimen for qualification of the produced loads. [Color figure can be viewed at wileyonlinelibrary.com].

A new multi‐axis loading fixture has been designed to analyze and determine fatigue in an implant under combined torque, bending, and compression. The details of the fixture are given in Figure 2, Table 1, and Appendix. The fixture can only be used in a test machine that is able to induce the load components of torque and compression independently. The fixture comprises three different parts in addition to the test specimen and there are two main pivot points. The test specimen is supposed to be attached in a rigid manner or in a manner as an implant is fixed to a bone in reality. Due to the fact that all real connections in a real fixture incur clearance as well as friction, the loading subjected to the test specimen during dynamic loading function will not correspond to an ideal kinematics model. Thus, the produced loading must be studied by using a calibration specimen and model experiments.

**Table 1:** Part List of the Invented Multi‐Axis Loading Fixture

Part	Material	Description
1	Steel	Proximal connection part
2	Steel	Distal connection part
3	Steel	Bending arm (machine connection)
4	Steel, titanium, coating	Bolts, screws, bearings
5	Composite	Bone replica (Sawbones®)
6	Steel	Distal support (machine connection)

It is important to note that the standard four‐point bending cannot induce any torsion or compressive axial loads (these are entirely lacking) and only bending performance could be compared with that of the multi‐axis fixture; this type of a study is available in the current literature.^8^

### Calibration Specimen

The system qualification was studied using a calibration specimen that imitated the tubular load‐carrying structure common in leg‐lengthening implants. In order to exclude implant‐specific effects, such as clearances and deformation of an internal lengthening machinery, a solid tube was selected. The wall thickness of the steel tube was 1.00–1.05 mm by measurement and the diameter 14.00–14.02 mm by measurement. The calibration specimen was instrumented using three strain gauges. Two axial gauges with a 5.0 mm grid length and temperature compensation for steel (KFG‐5‐120‐C1‐11L1M2R; Kyowa Electronic Instruments, Tokyo, Japan) were located at a ±90° circumferential offset in relation to the bone screw holes and aligned axially to match the specimen's longest dimension. A quarter‐bridge connection via the Wheatstone‐bridge was applied for the sensor‐system interface.

A shear‐strain gauge with a 2.0 mm grid length and temperature compensation for steel (KFG‐2‐120‐D31‐11L3M3R, Kyowa Electronic Instruments, Tokyo, Japan) was located at 0° circumferential offset in relation to the screw holes, and aligned with a 90° planar offset in relation to the specimen's longest dimension. A half‐bridge connection via the Wheatstone‐bridge was applied for the sensor‐system interface. This connection excludes any mutual resistance change difference (from the voltage signal) in the two at 45° positioned grids in the gauge in order to measure only the shear strain due to torque.

The axial positioning of the three gauges was 197 mm when measured from the proximal connection part, that is, the “upper corner” of the fixture. The calibration specimen was mounted to the test fixture using titanium bone screws, two per head, as shown in Figure 2. The entire fixture with a total length of 518 mm was fixed to the test machine using specific installation parts and the pivot moments were minimized via bearings.

### Measurement System and Test Program

The tests were performed using a testing machine (ElectroPuls E 3000; Instron, Norwood, MA) with a 3.0 kN load cell and computerized digital control (WaveMatrix; Instron, Norwood, MA). Strain readings from the strain gages were measured using the software Signasoft 6000 (Peekel Instruments, Gernsbach, Germany). The testing was performed in ambient conditions (22.7–23.2°C). The strain readings were recorded at 10 and 25 Hz frequencies depending on the load cycle rate per test. To analyze fatigue, a sinusoidal displacement waveform was applied with a targeted load ratio of the minimum and maximum load of *R* = 0.1. The ElectroPuls tester is capable of controlling independently torque and axial displacement (load) and the exact loading programs are given in Table 2.

**Table 2:** Test Programs for Multi‐Axis Loading Fixture With Calibration Specimen

Step	Load	Target Level/Limit
Quasi‐static torque		
Ramp	Torque	Until 6.2 Nm reached
	Compression	Until 0 N
Quasi‐static bending		
Ramp	Compression	Until −690 N reached
	Torque	Until 0 Nm
Quasi‐static combined loading		
Ramp	Compression +	Until −690 N reached
	Torque	Until 6.2 Nm reached
Cyclic torque		
Ramp	Torque	Limit 2.75° (mean)
Sinusoidal	Torque	Amplitude 2.25°
Cyclic bending		
Ramp	Compression	Limit −0.935 mm (mean)
Sinusoidal	Compression	Amplitude 0.765 mm
Cyclic combined loading		
Ramp	Compression +	Limit −0.935 mm (mean)
	Torque	Limit 2.75° (mean)
Sinusoidal	Compression +	Amplitude 0.765 mm amplitude 2.25°
	Torque	

After the above system qualification, the performance in practical force‐control tests was studied for the fixture. Dynamic combined sinusoidal loading (torque and compression subjected simultaneously) was subjected to the fixture at a load ratio of *R* = 0.1. The amplitude for torque was 2.79 Nm (leading to an average load of 3.41 Nm) and the peak load 6.2 Nm. The amplitude for compression was 310.5 N, which led to an average load of 379.5 N and the peak load 690 N.

### Analytical Transformation of Loads Into Strain Levels in an Implant Body

Analytical strain estimates were calculated to make the prognosis of the maximum strain levels based on typical quasi‐static loading subjected to a femur‐fixed implant, that is, as given in Table 2. First, the so‐called thin‐wall approximation for the torque component was applied so that the Bredt's equation was used to calculate the shear flow:

$$q=T/(2\pi r^{2})$$

where *T* is the torque and *r* is the (mid‐thickness) radius of the calibration specimen. The shear strain, γ, can be estimated using the shear modulus of steel (*G* = 76 GPa) and wall thickness (*t*):

$$\gamma =T/(t\cdot G\cdot 2\pi \;r^{2})$$

which here gives γ = 0.0003005 rad/rad.

The compressive axial strain was estimated as follows. First, the load introduction was presumed simplified for beam bending calculation. Pure axial strains were calculated using the cross‐sectional area (π[*r*_o_^2^−*r*_i_^2^]) and by applying the Young's modulus (*E* = 200 GPa) of the calibration specimen:

$$\varepsilon_{c}=F/\left(E\cdot \pi \cdot \left[r_{o}^{2}–r_{i}^{2}\right]\right)$$

where sub‐indices o and i refer to outer and inner radius of the tubular specimen, respectively. The stress due to the bending moment can be estimated using the classical beam bending theory. By calculating the second moment for a circumferential (hollow) cross‐sectioned beam as follows:

$$I=0.25\;\pi \;\left(r_{o}^{4}–r_{i}^{4}\right)$$

the axial strain due to the bending moment (*M*) only is given by:

$$\varepsilon_{b}=M\cdot y_{d}/(I\cdot E)$$

The total (maximum) axial strain can be calculated using the superposition simply by ε_axial_ = ε*_c_* ± ε*_b_*. By substituting a moment arm of 30 mm, and the mid‐radius for the bending distance (*y_d_*) we arrive to values of –0.00090 m/m (compressive side) and 0.000735 m/m (tensile side). The analytical estimate is clearly an approximation due to the presumed fully clamped pivot point. By estimating the deformation and the exact moment at the point of strain gauge (*x* = 197 mm) using equation:

$$M=M_{\text{proximal}}(1–x/L)$$

where *M*_proximal_ = *F* ∙ *y_d_* and *L* is the distance between pivot points, the prognosis can be made more accurate, giving values of –0.00057 m/m (compressive side) and 0.00040 m/m (tensile side).

### Finite Element Modeling and Load Condition Simulation

#### Finite Element Model Assembly

The entire test setup was modeled and simulated using Abaqus® 2017 (Dassault Systemes; Simulia, Velizy‐Villacoublay, France). An entire 3D model was generated to analyze the overall stress–strain field in the test system during the simulated loading. The main advantage of the finite element analysis (FEA) is that strains are analyzed all over the different parts, not only at one point like is the case with strain gauges. The loading and boundary conditions of the *proximal section* were given through a reference point located in the middle of the *bending arm* to run the FEA and analyze the loads in the calibration specimen.

**Figure 3:**
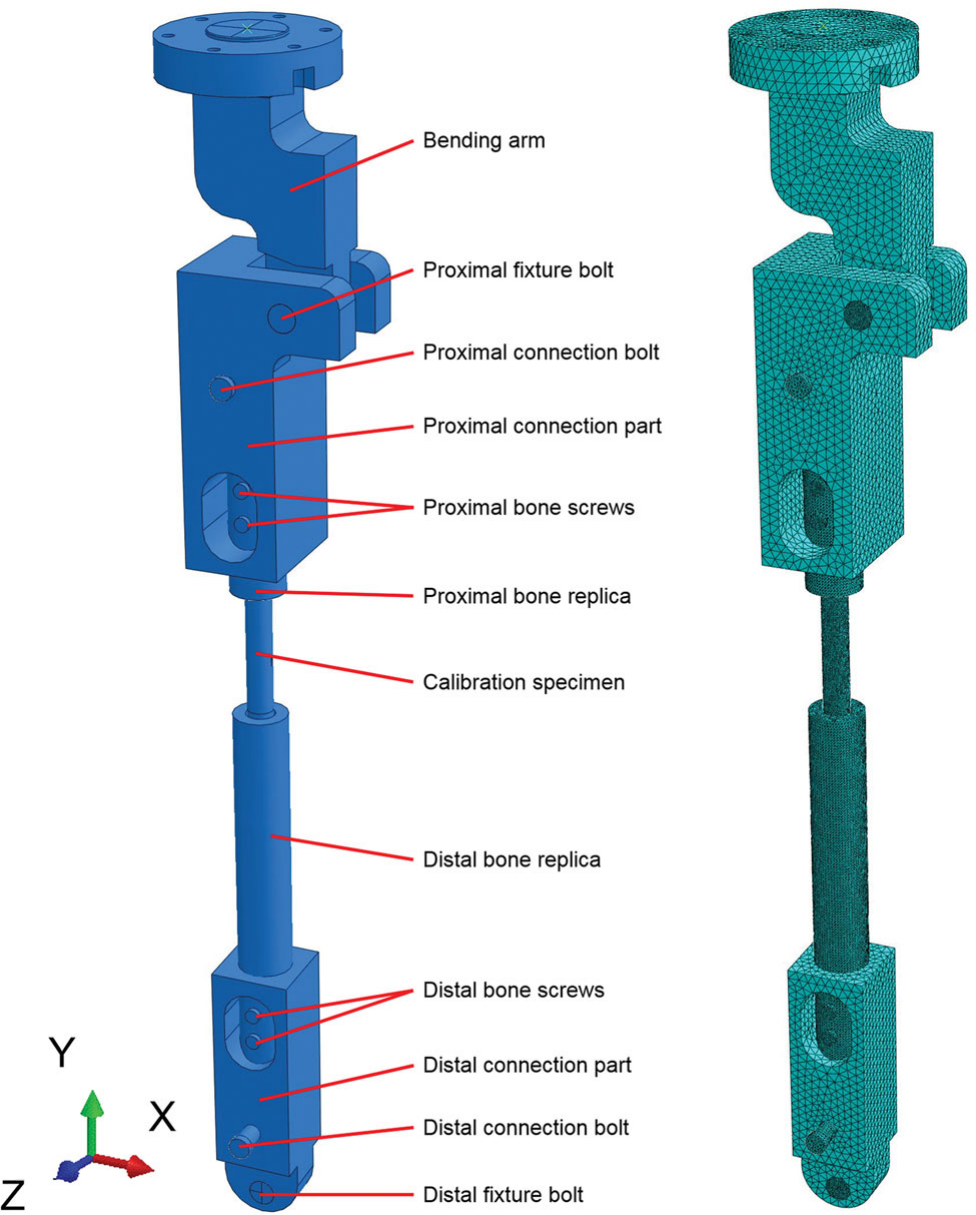
Finite element analysis (FEA) model of the fixture: separate parts and the finite element mesh. [Color figure can be viewed at wileyonlinelibrary.com].

In order to simplify the model, the *distal support* (test machine interface) was elided from the model, see Figure 3. Instead, restraining boundary conditions were set for the midline of the *distal fixture bolt*. The reference point was coupled (“coupling”) to the upper surface of the bending arm. In addition, the movement of the reference point in *X* and *Y* directions and rotation around *X* and *Y* axes were prevented to simulate the boundary condition of the testing scheme. The contact between the *proximal fixture bolt* and the bending arm as well as the bone replicas and the calibration specimen were modeled using surface‐to‐surface interaction at the contacts. All the other part connections were modeled using so‐called “tie constraint” connections. The material properties that were used in the model are listed in Table 3. All the parts used a linear‐elastic material model and were meshed with 10‐node quadratic tetrahedron elements (C3D10). The element size varied between 1 and 5 mm depending on the part. The overview of the element mesh is shown in Figure 3.

**Table 3:** Material Property Values Used in the Finite Element Model

Part	Material	*E* (GPa)	*ν* (–)
Fixture parts and connecting bolts	Steel	200	0.3
Calibration specimen	Steel	200	0.3
Bone replica	Short fiber‐filled epoxy	17^∗^	0.26^∗^
Bone screws	Titanium	114	0.3

All the materials were presumed isotropic and linear‐elastic for the analysis.

^∗^ Literature source: Simulated cortical bone (September 2018).^10^

#### Simulation Limitations

The solutions by the FE model of the test fixture and calibration specimen relies on the contact formulation on a FE basis, thus, the simulation does not include all air gaps and precise friction of the real test event. Therefore, the simulation results represent the working of an ideal, not worn‐out fixture with significant lubrication between different parts. It is important to note that the absolute results in this study refer to the calibration specimen. For a specific type of commercial, telescopic implant, a representative implant model must be created with a specific material data, for example, properties of certain metal alloy used in the implant body.

## RESULTS AND ANALYSIS

### Numerical Prognosis of the Strain Fields in the Calibration Specimen

**Figure 4:**
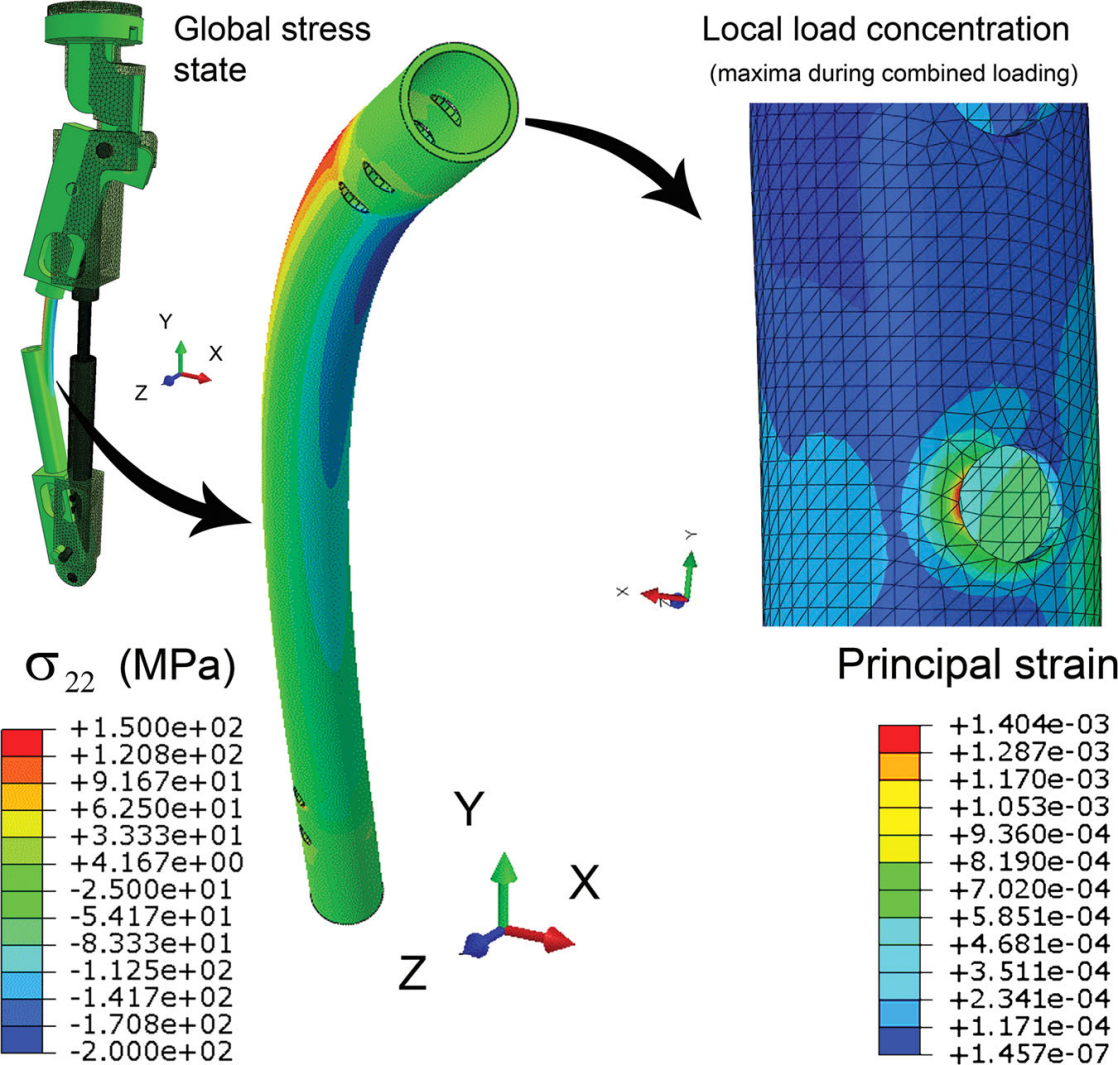
Finite element analysis of the axial stress (σ_22_) distribution in the calibration specimen and principal strains at a load concentration point (bone screw hole) under combined loading (axial load 6.2 kN, torque 6.2 Nm); in the magnified view only the calibration specimen is left visible. The deformation scale factor is set to ×23 for emphasizing the shape distortion in the figure. [Color figure can be viewed at wileyonlinelibrary.com].

Numerical, detailed FEA of the calibration specimen loading for the quasi‐static load condition gives the full stress–strain fields and reveals any peaking of stresses at stress concentrations. Analysis results for a combined loading case are shown in Figure 4. It can be seen that the axial stress peaks amidst the proximal screw holes, where the highest moment prevails. The peak strains, based on the Young's modulus for linear deformation, corresponded to strain levels of –0.001 m/m (compressive side) and 0.00075 m/m (tensile side) that are actually close to the conservative analytical estimation for a clamped pivot point. The FEA of the experimentation system for different loadings present exact prognoses to be compared with the strain gauge readings in experiments and will be supplemented in the following section.

### Quasi‐Static Loading Experiments as First‐Phase Qualification

**Figure 5:**
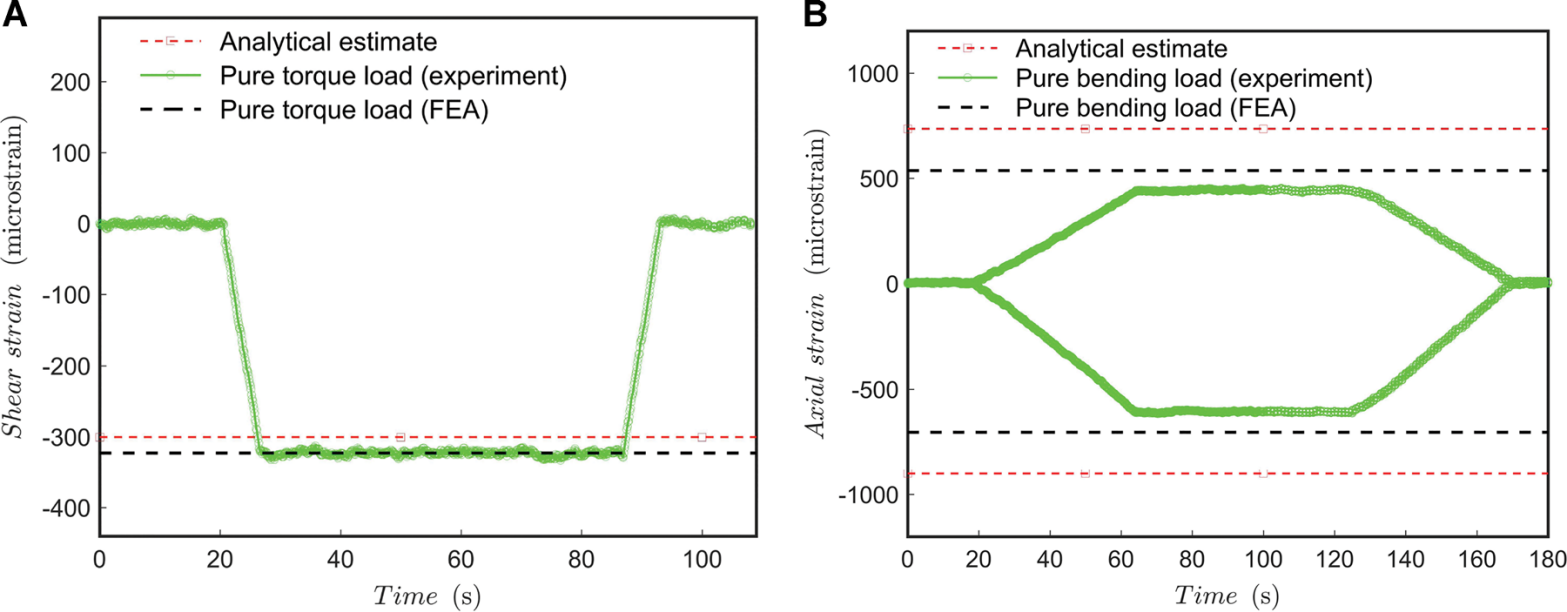
Quasi‐static test results. (A) Pure torque load (dwell 60 s); (B) pure bending load (dwell 60 s). [Color figure can be viewed at wileyonlinelibrary.com]

The measured shear strain, shown in Figure 5A, is rather exactly estimated and it keeps a constant value during a constant torque hold. The ratio of the measured shear strain to FEA was –0.000323 per –0.000326 = 0.99. The output of the compression test (pure bending exerted by compression, no torque) is shown in Figure 5B, and it can be seen that the measured axial strains are clearly lower than what was estimated by analytical methods (with the bending moment of the proximal head). The estimates by the FE simulation and the experiments are in an agreement—especially when noting that a numerical model is usually more rigid than the real system. The strains remain constant during a constant compressive hold. Anyhow, it seems that the multi‐axis fixture exerts less bending than anticipated: The ratio of the measured strain (compressive side) to the FEA estimate was –0.000606 per –0.000705 = 0.86. The ratio of the measured strain (tensile side) to the FEA estimate was 0.000444 per 0.000536 = 0.83.

**Figure 6:**
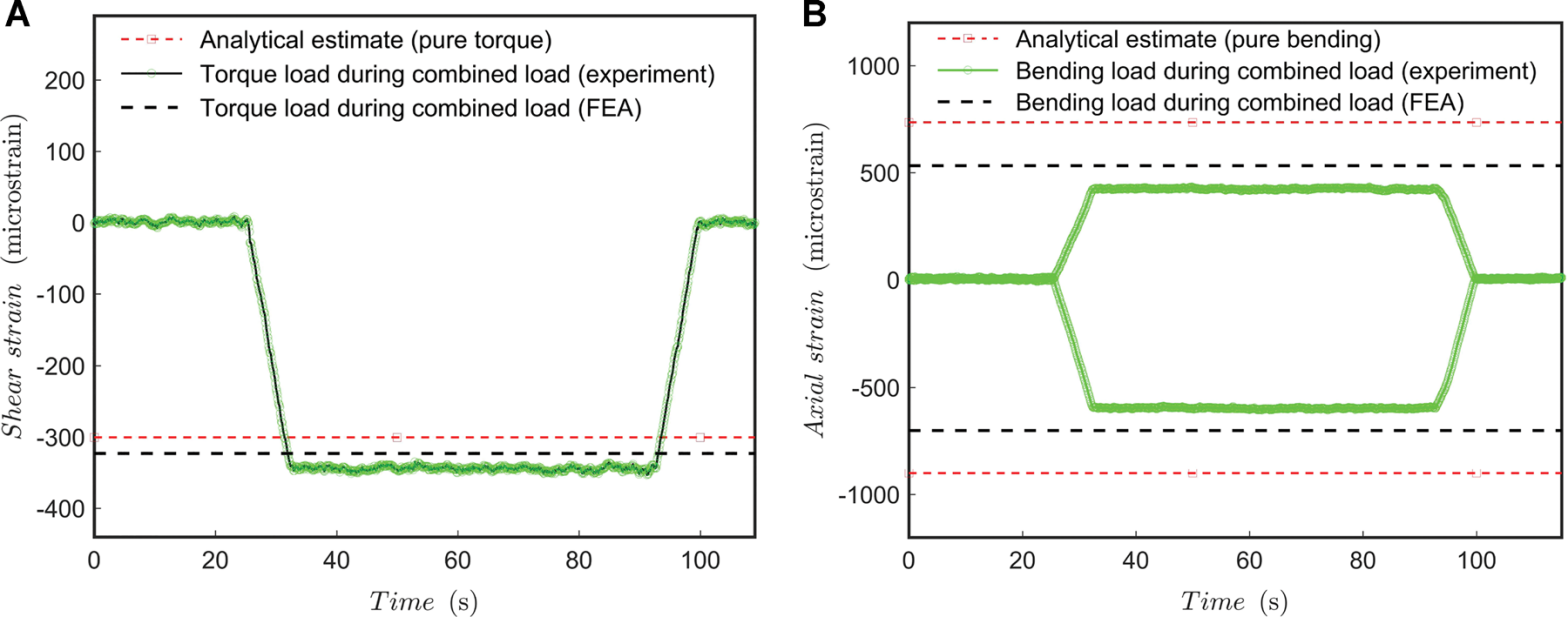
Quasi‐static combined load test results. (A) Torque load during combined loading (dwell 60 s); (B) bending load during combined loading (dwell 60 s). [Color figure can be viewed at wileyonlinelibrary.com]

The results of the combined loading test (bending and compression and torque) are shown in Figure 6, which indicate that slight torsion was subjected by the fixture during simultaneous compression—the effect is relatively low. For the axial strains, the results seem essentially corresponding to the pure compression test though the measured strains are perhaps slightly lower (1.3% lower in average). The accuracy of the predicted strains (FEA) in the combined load condition is of the order of predictions in the event of pure compression (Fig. 5B).

### Cyclic Loading Experiments in Displacement Control

**Figure 7:**
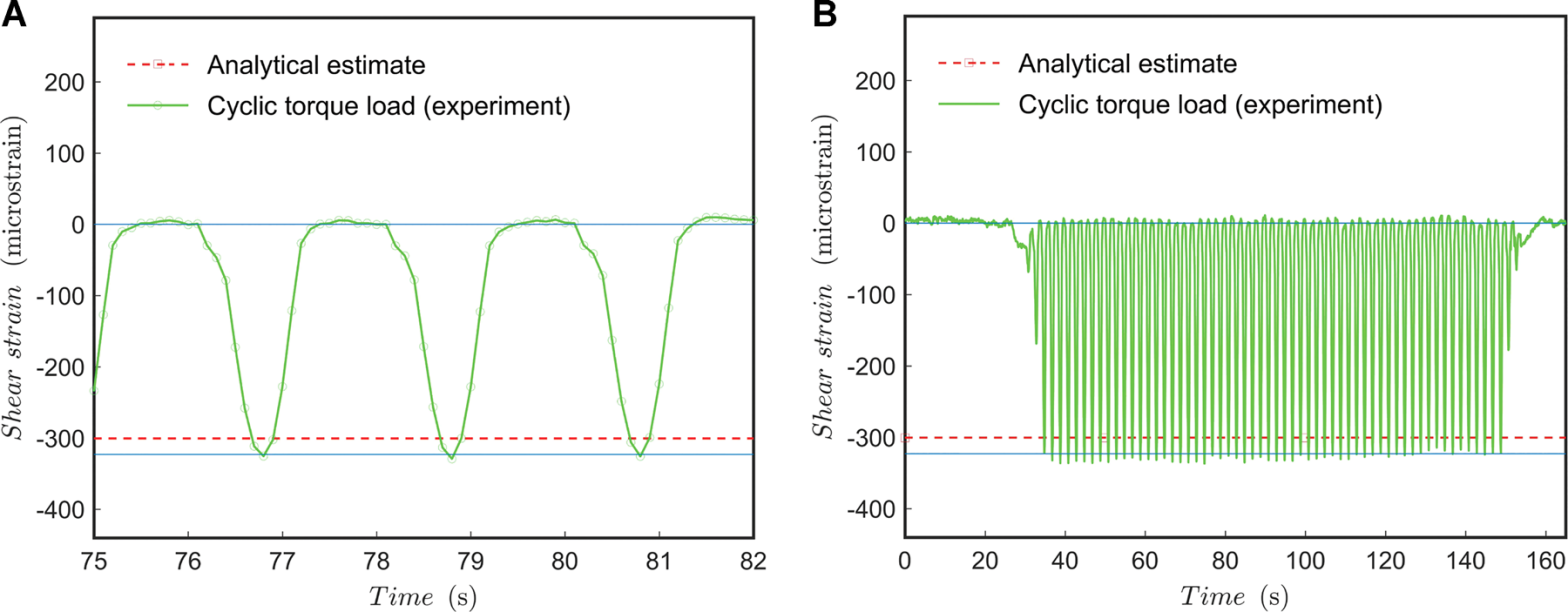
Cyclic load test for torque load. (A) Pure sinusoidal torque at 0.5 Hz; (B) pure sinusoidal torque (full spectrum, trend). Blue line added as a horizontal reference. [Color figure can be viewed at wileyonlinelibrary.com]

For separate dynamic loading tests, a sinusoidal (*A* ∙ sin(θ ∙ *t*)) displacement function per mode (torque and compression) was controlled by the testing machine. The angular velocity (rate of loading) was 0.5 Hz. The results for pure torque (2.75° mean displacement, 2.25° amplitude) in Figure 7 show that a torsional clearance between the fixture's parts induces discrepancy from a sinusoidal shape. Noting that the correct period (0.5 Hz) is achieved, the total specimen loading over time is somewhat non‐conservative because the system experiences near‐zero torque for a longer portion of the total test time. The peak value is rather well‐matched with the static torque tests. In the long run, there is a slight fluctuation, over a period of around 10 s, observable in the peak value and possibly a monotonic decrease in the peak value. The fluctuation can be assumed negligible but the decrease in the peak value should be studied during longer (e.g., 1–2 million cycles) test runs whenever a long‐term implant usage is of interest.

The results for bending (–0.935 mm mean displacement and 0.765 mm amplitude) indicated that bending clearances induce discrepancy from the sinusoidal shape. Hence, the total specimen loading over time is slightly non‐conservative because the system experiences near‐zero bending for a longer portion of the total test time. However, the effect of the longer zero‐load portion is negligible for materials with no creep over the applied load range, such as those of traditional implant alloys. For pure bending, the peak values are surprisingly well‐matched with the static tests and are close to the numerical estimates too.

**Figure 8:**
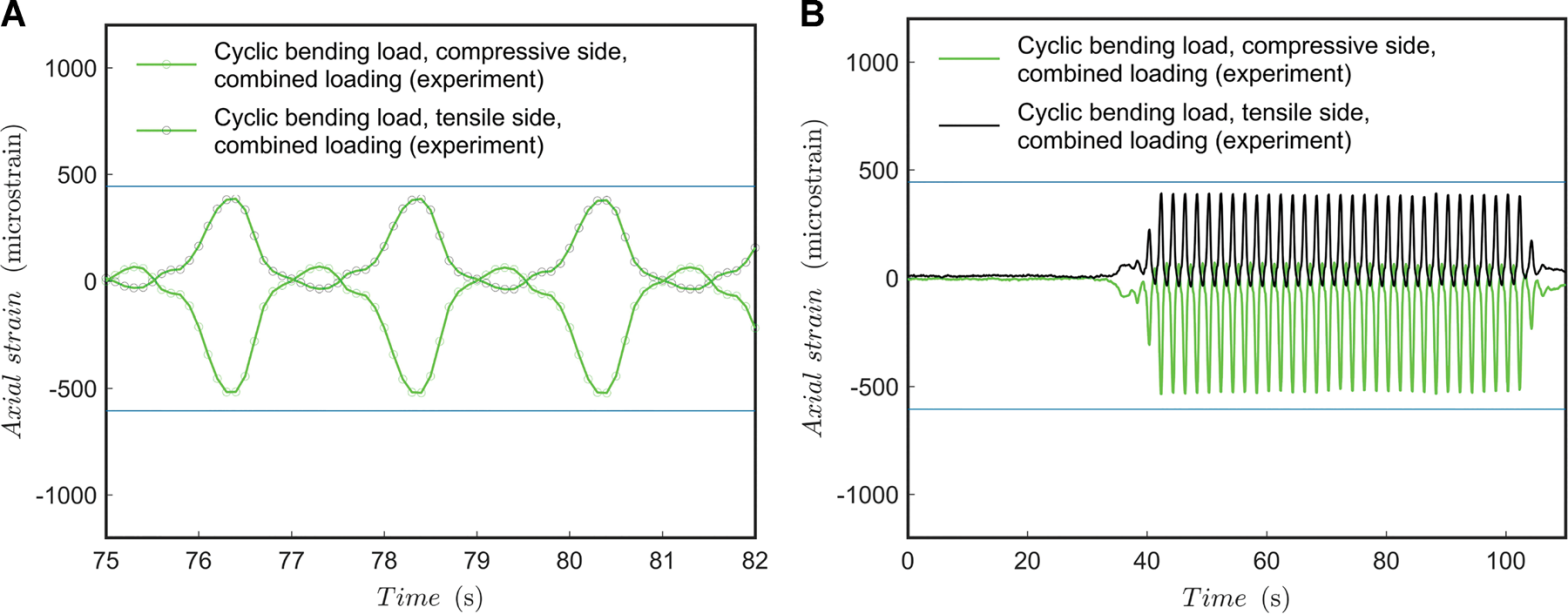
Cyclic load test for combined load at 0.5 Hz. (A) Axial strain during testing; (B) axial strain trend. Blue line added as a horizontal reference. [Color figure can be viewed at wileyonlinelibrary.com]

**Figure 9:**
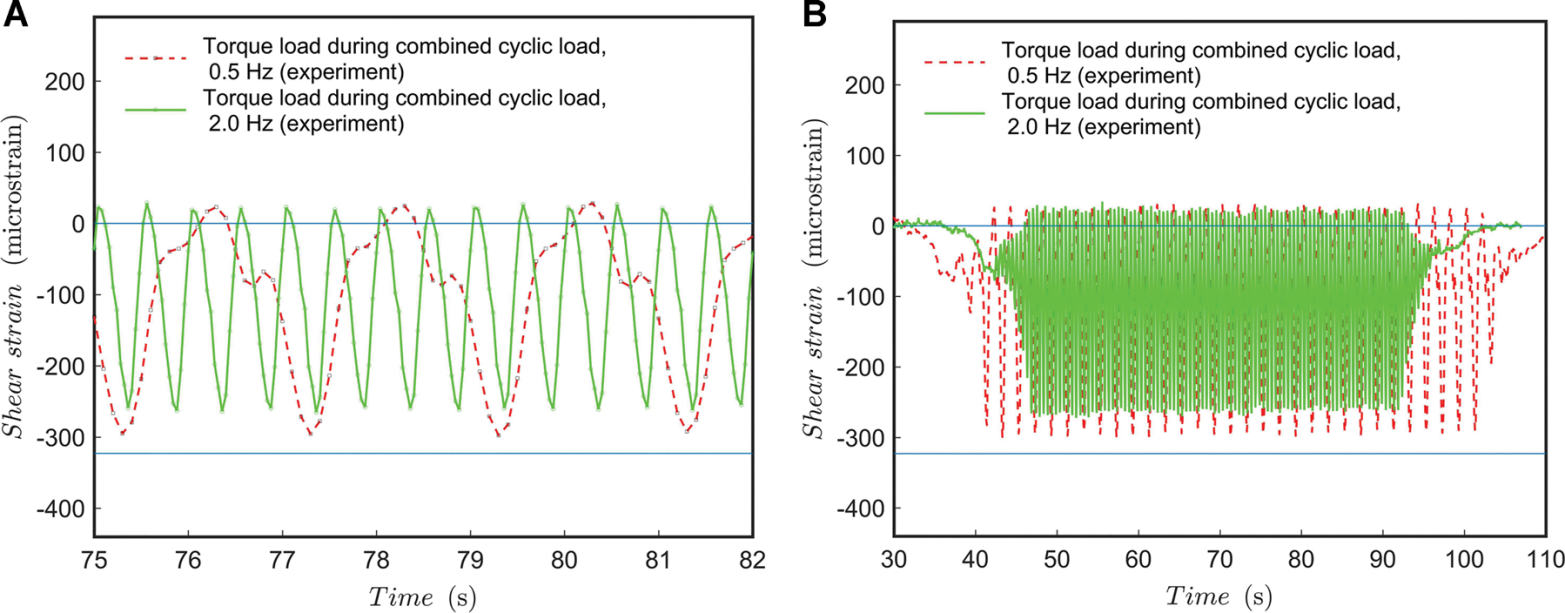
Cyclic load test for combined load at 2.0 Hz with comparison to 0.5 Hz test. (A) Shear strain during testing; (B) shear strain trend. Blue line added as a horizontal reference. [Color figure can be viewed at wileyonlinelibrary.com]

**Figure 10:**
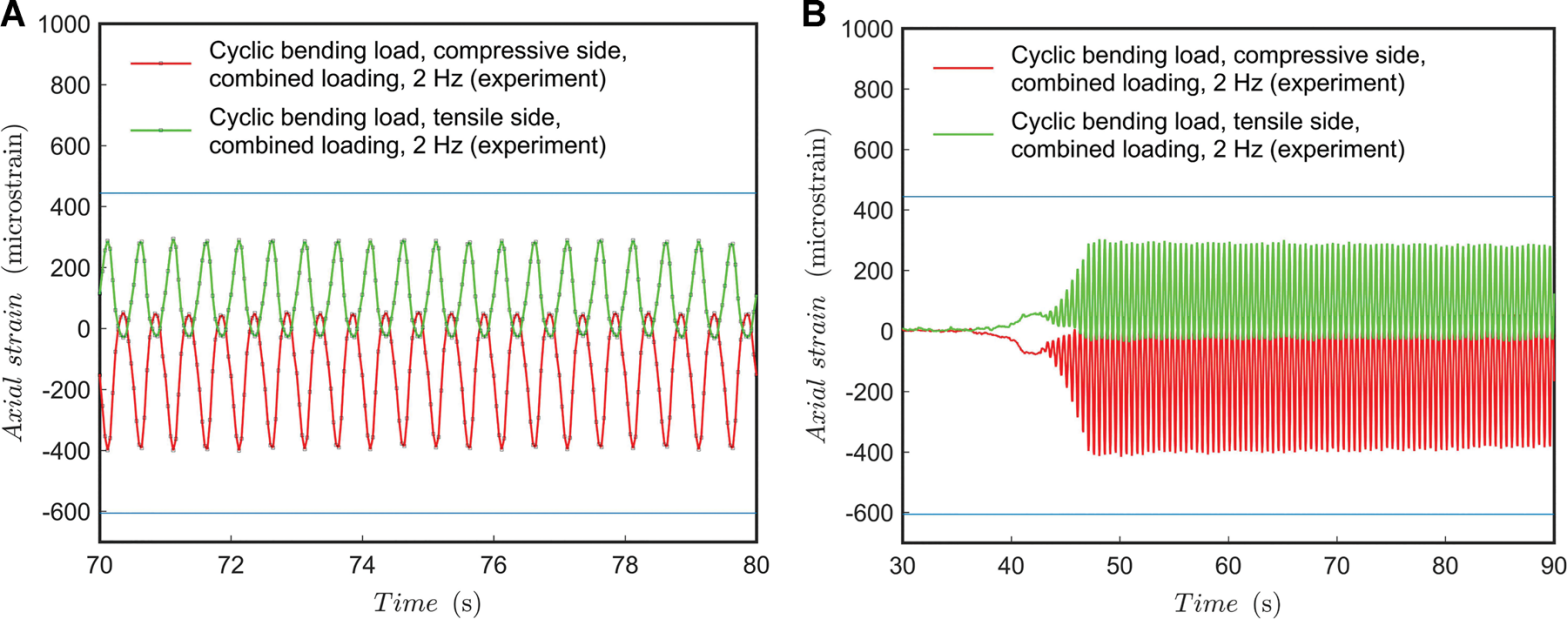
Cyclic load test for combined load at 2.0 Hz. (A) Axial strain during testing; (B) axial strain trend. Blue line added as a horizontal reference. [Color figure can be viewed at wileyonlinelibrary.com]

For combined dynamic loading tests, a sinusoidal (*A* ∙ sin(θ ∙ *t*)) function per mode (torque and compression) was controlled by the testing machine. The angular velocity, defining the rate of loading, was 0.5 and 2 Hz. The results for 0.5 Hz are shown in Figures 8 and 9 and the results for 2 Hz rate are shown in Figures 9 and 10.

The results show that the shape discrepancy compared with an ideal sinusoidal form is offset from the lowest load point (compared with separate dynamic loading tests). It should be noted that the test machine's control was not phase‐synchronized, and the highest loading points (peaks) do not appear at the same time for shear and axial load in the test specimen. Probably, the phase‐difference in the test machine control affects the location of the shape discrepancy from the sinusoidal shape in the load‐axis direction. However, this assumption should be validated by further tests. In any case, the total loading is somewhat non‐conservative (the system experiences lower loads for longer periods per cycle). Also, what is important to note, is that the shear and axial strains are occasionally zero and even change signs at peaks—this should not be possible by the given test machine control function. This can be a problem of machine control or change of pivot clearance during testing with pure displacement control yet reveals the effect of accumulation of error factors in a multi‐axis loading scheme due to the inevitable clearances between fixture parts.

The results show that the shape discrepancy compared to an ideal sinusoid seems less when the rate of loading was set to 0.5 Hz and compared to 2 Hz. However, the amount of data points (given by recording frequency) might be too a low for observing the actual performance. Again, strain values change sign, which should not be possible by the given control function. What is important to note, at a 2 Hz rate of loading, is that the peak loads are lower—for both torque and bending load, the test specimen experiences clearly less mechanical exertion. For longer periods, there is also monotonic decrease observable in the peak strain values. The decrease in the peak value could be studied during longer test runs—it is presumed to be due to a small drift at screw‐hole joints and the subsequent increase of clearance. Due to the increased clearance, fixture speed and inertia loads are higher per cycle and lead to higher contact friction at joints lowering the forces experienced by the test specimen. This might be of interest of a designer if several millions of load cycles are considered for an implant.

### Cyclic Loading Experiments in Force‐Controlled Mode

**Figure 11:**
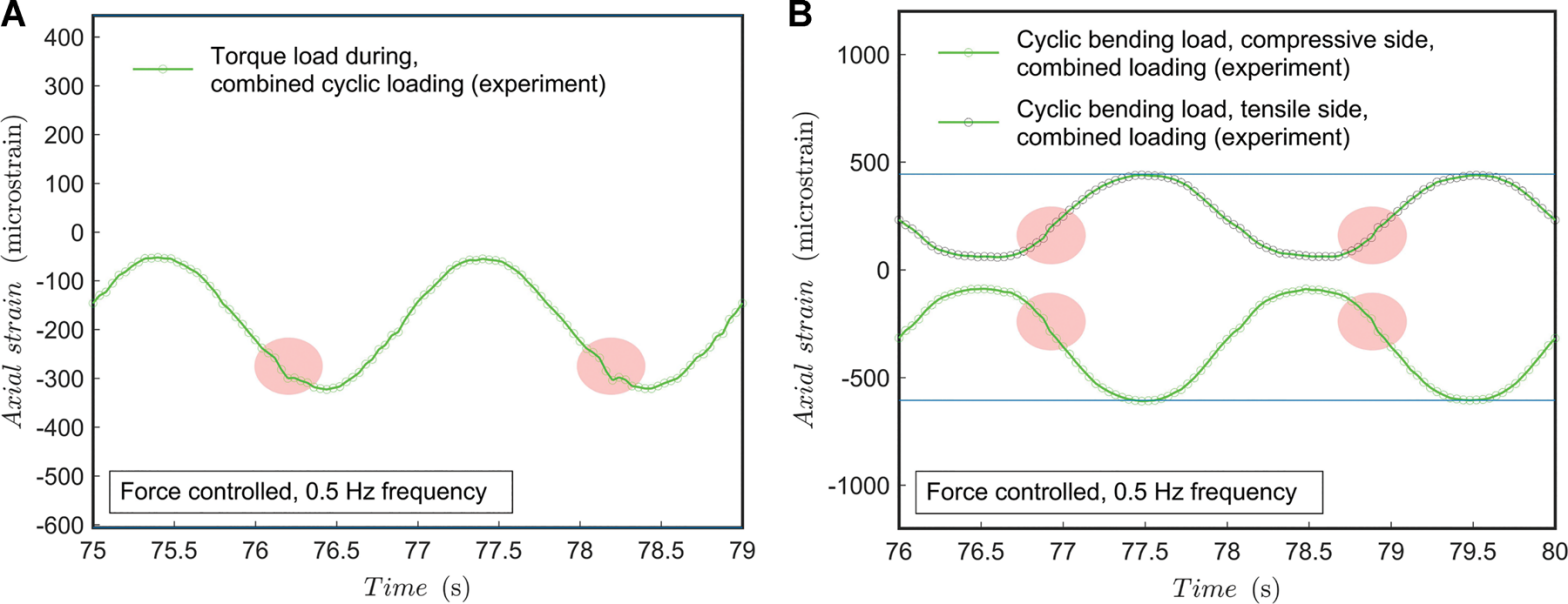
Cyclic load test for combined load at 0.5 Hz in a force‐controlled mode of the test machine. (A) Shear strain during testing; (B) axial strain during testing. The points of discrepancy emphasized by red shadow. Blue line added as a horizontal reference. [Color figure can be viewed at wileyonlinelibrary.com]

For the force–torque control experiments, a sinusoidal (*A* ∙ sin(θ ∙ *t*)) force–torque function per component, referring to torque and compression, was controlled by the testing machine. Amplitude and mid‐point (average loading) was adjusted to a load ratio of *R* = 0.1. The results for the loading rate of 0.5 Hz are shown in Figure 11. Interestingly, the torque exceeds slightly the targeted level yet the targeted *R*‐ratio is rather well achieved. The shape discrepancy is clearly less than for the displacement‐controlled test results—as is typical for force‐control. The shape discrepancy for torque occurs at high loads, which implies that the reason might not be rotational clearance due to slag between parts but the interaction with the zeroing and respective clearance in the axial direction. It should be noted that the compression and torque are off‐phase, so that the minimum bending occurs about at the highest torque load with approximately 1‐s phase‐difference. In the long run, the peak values remain relatively well constant and no significant fluctuation or shift emerges.

## DISCUSSION

### Multi‐Axis Cyclic Test Procedure

**Figure 12:**
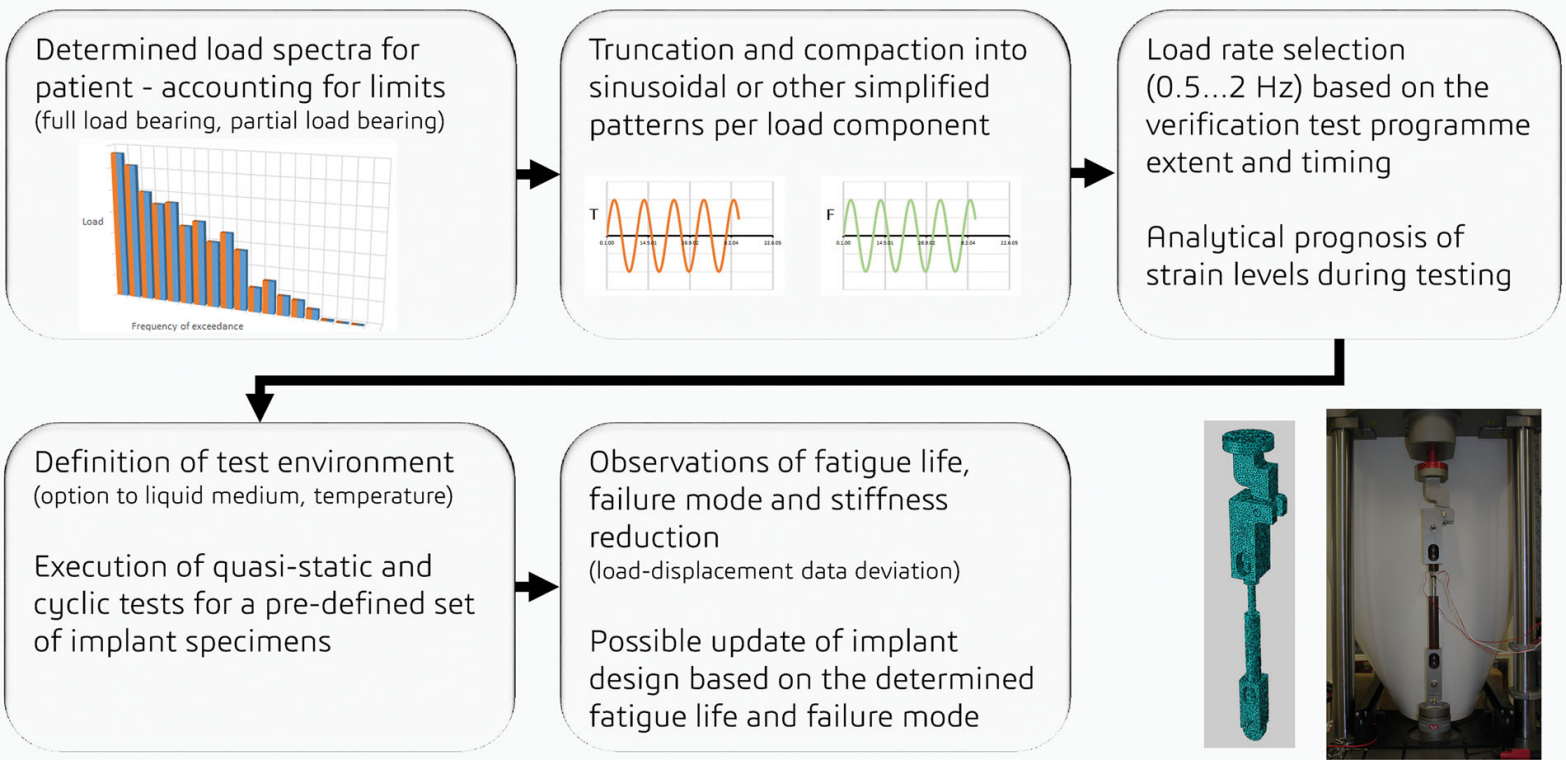
The concept of fatigue life determination procedure for intramedullary tubular and solid nail implants subjected to multi‐axial loads by using the multi‐axis test fixture. [Color figure can be viewed at wileyonlinelibrary.com]

This work presents a multi‐axis test fixture and test procedure for determining fatigue limits for intramedullary and solid nail implants when subjected to multi‐axial loadings. The correct application of the method requires understanding the operation limits of the test fixture in terms of loading rate and accuracy of the reproduced load sequences. The study presents the behavior of the fixture‐implant coupling during quasi‐static loading and cyclic loading—analyzed here for single and multi‐axial loadings. Additionally, 0.5 Hz and 2 Hz load rates were studied to discover load rate dependency. On the basis of the results presented and the traditions of the field, the procedure in the form of a flow‐chart, as illustrated in Figure 12, is defined for multi‐axial cyclic testing.

### Typical Reported Peak Loads and Implant Design Loads

The value of the reported axial compressive force used for the implant design for different telescopic intramedullary implants ranges through 223–4,000 N.^3^, ^5^ As a note, the full load‐bearing capability during slow walking would be the best practice for any leg implant; this means that 100% of the body weight and inertia is subjected to the nail‐bone system. The full load‐bearing would lead to force values of approximately 2–3 times the body weight.

Natural muscle tonus mainly induces a compressive axial load with respect to the nail long axis. This compressive load must be overcome by the power unit during the active lengthening stage, that is, during distraction. For tibia, the required force during distraction can reach a level of 340 N; the highest force is usually required at the end of the lengthening stage.^11^ The scientific literature about the distraction forces for femur implants is scarce. For a circular lengthening fixator, a maximum of 673 N has been reported for a child's femur.^12^ For the Fitbone® concept,^2^, ^13^ the power unit can reach a peak force of 1,800 N, which has been reported to be twice as high as what is needed for femur.^14^ For the ISKD concept, a maximum distraction load of 1,400 N has been reported for bench testing purpose.^15^

Transient loads are highly dependent on the type of body movement. In general, the decrease in the time scale leads to higher (dynamic) loads. Therefore, only a quasi‐static load‐bearing can be allowed, that is, patient standing and slow transfer of weight from one leg to another (i.e., slow walking). The dynamic loading of the nail structure and the related verification tests are typically referred to as fatigue testing and carried out by subjecting a cyclic load to the implant structure.^9^, ^16^ The femur and tibia‐implanted specimens are loaded by a full 3D load condition during patient movement. Thus, axial compression and out‐of‐plane shear forces, as well as bending and torsion, are loading the bone concurrently. The combination of axial compression, bending moments and torsion tend to cause the main challenges for the structural integrity of implanted structures. The strain gauge measurements have previously been used by Taylor et al.^17^, ^18^ to determine in vivo loads in the mid‐section of the femur. In these studies, strain data was collected from patients whose knee and part of the femur was replaced^17^ whereas Schneider et al.^19^ used intramedullary nail, which was implanted inside a multi‐fragmented femur. All these measurements showed significant values of loads during different standing positions and daily activities. The research by Taylor et al.^18^ showed the graphs of the loads during a gait cycle—the peaks of different loads occur approximately at the same instance during the gait cycle. Another approach for defining the loads in femur has been the musculo‐skeletal modeling: Duda et al.^20^ formed a model to calculate the loads in femur during the walking conditions. The results including the activity of every muscle showed clear variation in loads, which was depending on the gait progress and location on the femur. The introduction of in vivo hip contact loads of four patients to the model gives wider perspective to the variation in the loading among individuals.^21^ Simulations and measurements^22^, ^23^ suggest similar behavior for tibia—significant values for axial force, bending moments and torque during a gait cycle

### Supplementary Use of the Multi‐Axis Fixture

In the current literature, the importance of the length and location of osteotomy on the loads acting on an implant structure have been clearly emphasized.^24^ Here, the location of the osteotomy can be simulated by using the multi‐axis fixture and by changing the length of the bone replica tubes (part number five, Fig. 2). The position of the “gap” simulating the osteotomy location is important for intramedullary leg‐lengthening implants due to their inherent telescopic structure. Typically, the less stiff part of the telescopic structure is fixed inside normal bone in order to be outside the developing bone during the consolidation phase,^25^ where the loads and fatigue are the most stringent. Finite element models can be valuable tools to estimate alternative materials for implant devices. Eventually, the significant engineering simplifications—necessary for practical models^26^—can only be validated through experiments of representative combined loadings. The multi‐axis fixture investigated in this work can offer a more accurate loading condition representative of that during patient motion and support modeling work alongside its primary application of fatigue testing of nails as the required pre‐clinical phase.

## CONCLUSIONS

This study introduces an alternative test fixture and procedure to standard (see ASTM F1264) activities for extending the testing of intramedullary leg‐lengthening implants with a representative, simultaneous combination of compression, bending, and torque. The fixture requires a test machine capable of independent control of torque and axial force. A tubular calibration specimen instrumented using strain gauges was used to examine the loads that are subjected to the test specimen. Single and multi‐component loadings were considered, as well as static and dynamic loadings. The results of the calibration test program are concluded as follows:

- The multi‐axis test fixture can be used to create a very accurate set of compression, bending, and torque during a quasi‐static test for the design of intramedullary implants;
- The clearances between the fixture induce discrepancy in the sinusoidal load shape during 0.5…2 Hz cyclic loading tests due to accumulated freedom and frictional effects;
- Force‐controlled cyclic tests produce accurately the sinusoidal load shape and reproduce load cycles without drift during long‐term tests, when the load rate (frequency) is kept below 2 Hz.

## AUTHORS’ CONTRIBUTION

K.M.: Substantial contributions to research design, analysis of data, drafting the paper; P.T.: Substantial contributions to research design, analysis of data, drafting the paper; J.J.: Substantial contributions to research design, analysis of data; H.J.: Substantial contributions to research design, revising paper critically; R.A.: Substantial contributions to research design, revising paper critically; S.D.: Substantial contributions to research design, revising the paper critically, approval of the submitted version.

## References

[1] Guichet J‐M , Casar R. 1997 Mechanical characterization of a totally intramedullary gradual elongation nail. Clin Orthop 337:281–290. 10.1097/00003086-199704000-00032 9137201

[2] Baumgart R , Burklein D , Hinterwimmer X. 2005 The management of leg‐length discrepancy in Ollier's disease with a fully implantable lengthening nail. J Bone Joint Surg 87:1000–1004. 10.1302/0301-620X.87B7.16365 15972921

[3] Thonse R , Herzenberg J , Standard S , et al. 2005 Limb lengthening with a fully implantable, telescopic, intramedullary nail. Oper Tech Orthop 15:355–362.

[4] Paley D. 2015 Precice intramedullary limb lengthening system. Expert Rev Med Devices 12:231–249. 25692375 10.1586/17434440.2015.1005604

[5] Reynders P , Van Bael J , Peter‐John M , et al. 2008 The hydraulic lengthening nail for lengthening the lower limb. A prospective cohort trial of thirty‐four nails. Injury Extra 39:163–164.

[6] Tiefenboeck T , Zak L , Bukaty A , et al. 2016 Pitfalls in automatic limb lengthening—first results with an intramedullary lengthening device. Orthop Traumatol Surg Res 102:851–855. 27527249 10.1016/j.otsr.2016.07.004

[7] Limmahakhun S , Oloyede A , Sitthiseripratip K , et al. 2017 Stiffness and strength tailoring of cobalt chromium graded cellular structures for stress‐shielding reduction. Mater Des 114:633–641.

[8] Kanerva M , Besharat Z , Livingston R , et al. 2015 Plasticity effects during the 4‐point bending of intramedullary leg lengthening implants with telescopic structures. In: Proceedings of the 6th international conference on mechanics and materials in design. Ponta Delgada, Portugal. July 26–30.

[9] ASTM F 1264‐16e1 . 2018 Standard specification and test methods for intramedullary fixation devices. West Conshohocken, PA: ASTM International.

[10] Simulated cortical bone. September 2018. Material selection quide . Available from: https://www.sawbones.com/biomechanical/material-selection/

[11] Brunner U , Cordey J , Schweiberer L , et al. 1994 Force required for bone segment transport in the treatment of large bone defects using medullary nail fixation. Clin Orthop 301:147–155. 8156665

[12] Younger A , Mackenzie W , Morrison J. 1994 Femoral forces during limb lengthening in children. Clin Orthop 301:55–63. 8156697

[13] Betz A , Hax P‐M , Hierner R , et al. 2008 Läangenkorrekturen der unteren extremität mit voll implantierbaren distraktionsmarknägeln. Trauma Berufskrankh 10:45–54.

[14] Wolfson N , Hearn T , Thomason J. 1990 Force and stiffness changes during Ilizarov leg lengthening. Clin Orthop 250:58–60. 2293945

[15] Cole JD , Justin D , Kasparis T , et al. 2001 The intramedullary skeletal kinetic distractor (ISKD): first clinical results of a new intramedullary nail for lengthening of the femur and tibia. Injury 4:SD129–39. 10.1016/s0020-1383(01)00116-4 11812486

[16] ISO 7206‐4 . 2010 Implants for surgery partial and total hip joint prostheses part 4. Geneva, Switzerland: International Organization for Standardization.

[17] Taylor S , Walker P. 2001 Forces and moments telemetered from two distal femoral replacements during various activities. J Biomech 34:839–848. 11410168 10.1016/s0021-9290(01)00042-2

[18] Taylor SJG , Walker PS , Perry JS , et al. 1998 The forces in the distal femur and the knee during walking and other activities measured by telemetry. J Arthroplasty 13:428–437. 9645524 10.1016/s0883-5403(98)90009-2

[19] Schneider E , Michel MC , Genge M , et al. 2001 Loads acting in an intramedullary nail during fracture healing in the human femur. J Biomech 34:849–857. 11410169 10.1016/s0021-9290(01)00037-9

[20] Duda G , Schneider E , Chao E. 1997 Internal forces and moments in the femur during walking. J Biomech 30:933–941. 9302616 10.1016/s0021-9290(97)00057-2

[21] Heller MO , Bergmann G , Deuretzbacher G , et al. 2001 Influence of femoral anteversion on proximal femoral loading: measaurement and simulation in four patients. Clin Biomech 16:644–649. 10.1016/s0268-0033(01)00053-5 11535345

[22] Wehner T , Claes L , Simon U. 2009 Internal loads in the human tibia during gait. Clin Biomech 24:299–302. 10.1016/j.clinbiomech.2008.12.007 19185959

[23] Orthoload . 2018 Loading of orthopaedic implants. Database. October, 2018. Available from: https://orthoload.com/

[24] Okyar A , Bayoglu R. 2012 The effect of loading in mechanical response predictions of bone lengthening. Med Eng Phys 34:1362–1367. 22858057 10.1016/j.medengphy.2012.07.007

[25] Calder P , Laubscher M , Goodier W. 2017 The role of intramedullary implant in limb lengthening. Injury 48:S52–S58. 28449859 10.1016/j.injury.2017.04.028

[26] Samiezadeh S , Tavakkoli Avval P , Fawaz Z , et al. 2014 Biomechanical assessment of composite versus metallic intramedullary nailing system in femoral shaft fractures: a finite element study. Clin Biomech 29:803–810. 10.1016/j.clinbiomech.2014.05.010 24951320

